# No evidence of Zika, dengue, or chikungunya virus infection in field-caught mosquitoes from the Recife Metropolitan Region, Brazil, 2015

**DOI:** 10.12688/wellcomeopenres.15295.1

**Published:** 2019-06-10

**Authors:** Anita Ramesh, Claire L. Jeffries, Priscila Castanha, Paula A. S. Oliveira, Neal Alexander, Mary Cameron, Cynthia Braga, Thomas Walker

**Affiliations:** 1 Department of Parasitology, Instituto Aggeu Magalhães(IAM/FIOCRUZ Pernambuco), Recife, Brazil; 2 Department of Infectious Disease Epidemiology, London School of Hygiene & Tropical Medicine, London, WC1E 7HT, UK; 3 Department of Disease Control, London School of Hygiene & Tropical Medicine, London, WC1E 7HT, UK; 4 Department of Virology, Instituto Aggeu Magalhães(IAM/FIOCRUZ Pernambuco), Recife, Brazil; 5 Universidade Estadual de Pernambuco (UPE), Recife, Brazil

**Keywords:** Zika virus, dengue virus, chikungunya virus, Aedes aegypti, Culex quinquefasciatus, molecular xenomonitoring, arboviruses, neglected tropical diseases, disease surveillance, urban areas

## Abstract

**Background: **The Recife Metropolitan Region (RMR), north-eastern Brazil, was the epicentre of the 2015 Zika virus (ZIKV) epidemic, which was followed by a 2016 chikungunya virus (CHIKV) epidemic. It historically has amongst the highest incidence of dengue virus (DENV) infections and is the only remaining focus of lymphatic filariasis (LF) in Brazil. In early 2015, a molecular xenomonitoring surveillance project focused on *Culex (Cx.) quinquefasciatus* commenced to inform LF elimination activities. *Aedes (Ae.) aegypti* mosquitoes were also collected, concurrent with the first microcephaly cases detected in the RMR. In terms of the 2015 ZIKV epidemic, these are the earliest known field-collected mosquitoes, preserved for potential RNA virus detection, when ZIKV was known to be circulating locally.

**Methods:   **Adult mosquitoes were collected in two sites (0.4 km ^2^) of Sítio Novo, Olinda, RMR, from July 22 to August 21, 2015. Mosquitoes were morphologically identified, sorted by physiological status, and pooled (up to 10 mosquitoes per house per day or week). RNA was extracted, reverse transcribed and the cDNA tested by real-time PCR.

**Results: **A total of 10,139 adult female *Cx. quinquefasciatus* and 939 adult female *Ae. aegypti* were captured. All female *Ae. aegypti* specimens were included within 156 pools and screened for ZIKV, DENV and CHIKV. In addition, a sub-set of 1,556 *Cx. quinquefasciatus* adult females in 182 pools were screened for ZIKV. No evidence of infection with any of the three arboviruses was found.

**Conclusions: **The absence of arbovirus detection may have been expected given the extremely restricted geographic area and collection of mosquitoes during a very short time period of peak mosquito abundance (July–September), but low arbovirus circulation (November–March).  However, this study demonstrates the potential to retrospectively screen for additional unexpected pathogens in situations of rapid emergence, such as occurred during the outbreak of ZIKV in the RMR.

## Introduction

Vector-borne diseases (VBDs), which according to the World Health Organization (WHO) constitute nearly 20% of all communicable diseases, exert an enormous health, health system, and economic toll worldwide ^1, 2^. VBDs transmitted by the mosquitoes *Aedes (Ae.) aegypti* (e.g., dengue, chikungunya, Zika) and *Culex (Cx.) quinquefasciatus* (e.g., lymphatic filariasis), which thrive in crowded cities, are particularly difficult to control in areas of rampant urbanization. This is especially the case in a country like Brazil which, according to the United Nations, is over 86% urbanized ^3^.

Zika virus (ZIKV) and dengue virus (DENV) (family *Flaviviridae*, genus *Flavivirus*), as well as chikungunya virus (CHIKV) (family *Togaviridae*, genus *Alphavirus),* are single-stranded positive-sense RNA viruses, which are largely transmitted by vector species within the *Aedes* genus of mosquitoes. In Brazil, as in much of the rest of the world, *Ae. aegypti* is the principal vector of these arboviruses in urban areas, whereas *Ae. albopictus* is the principal vector in peri-urban and rural areas ^4^. Recent evidence exists that, in addition to the traditionally accepted mosquito-borne transmission pathways, sexual transmission of ZIKV can also occur ^5– 7^.

In Brazil, DENV was first introduced in the 16 ^th^ century, causing sporadic epidemics over subsequent centuries, but disappeared alongside *Ae. aegypti* (targeted in yellow fever elimination campaigns) for several decades in the mid-20 ^th^ century ^4, 8, 9^. By the 1980s, DENV had re-emerged with the re-infestation of *Ae. aegypti*, and has since been a serious public health problem, with increasing morbidity and mortality ^10^. By 2015, the economic impact of dengue, from a societal perspective, was estimated to be $1.2 billion USD per year ^11^.

After its discovery in 1947 in the Zika Forest of Uganda, ZIKV was largely restricted to Africa and Asia for nearly 60 years and was considered to cause relatively mild morbidity and low mortality in humans ^7, 12^. In 2007, however, a ZIKV outbreak on the Yap Islands quickly spread to French Polynesia and other Pacific Islands over the next eight years ^13^. The geographical spread of ZIKV appeared similar to that of DENV and CHIKV, more severe neurological complications began to be associated with ZIKV infections, and reports from this time indicated a potential association between ZIKV infection and Guillain–Barré syndrome ^7, 14– 17^. 

In December 2014, the north-eastern Brazilian state of Pernambuco reported several cases of a rash-associated illness and in March 2015 the states of Rio Grande do Norte and Bahia confirmed the first clinical isolates associated with such illnesses to be ZIKV ^18– 21^. By July 2015, ZIKV had been detected in 12 Brazilian states and by August 2015, health authorities in Pernambuco reported a sharp increase in microcephaly cases that were soon suspected to be associated with ZIKV infections ^20, 22^. On November 11, 2015 the Brazilian Ministry of Health declared Zika and microcephaly to be a national public health emergency ^23^. On December 1, 2015 the Pan American Health Organization (PAHO)/WHO reported the possible association between ZIKV and microcephaly in the Americas and provided public health guidelines for case management, surveillance, and vector control ^24^. On February 1, 2016, the WHO declared ZIKV to be a Public Health Epidemic of International Concern (PHEIC) ^25^. However, by this time, CHIKV, rather than ZIKV, had become the predominantly circulating arbovirus in the Recife Metropolitan Region (RMR) and Brazil in general ^26^.

The RMR has a population of over 3.7 million people in 14 municipalities, including the cities of Recife and Olinda ^27^. Nearly 30% of RMR residents live in *favelas* (slums) and areas of suboptimal municipal infrastructure, including proximate to polluted water bodies and in areas lacking refuse collection, which provide optimal conditions for *Cx. quinquefasciatus* and *Ae. aegypti* proliferation ^28^. By 2018, many RMR municipalities had formally completed over 15 years of mass drug administration (MDA) via the WHO-led Global Programme to Eliminate Lymphatic Filariasis (GPELF) ^29, 30^. Nevertheless, the RMR is not only the last focus of lymphatic filariasis (LF) in Brazil, it is also largely considered to be the epicenter of the 2015-2016 ZIKV epidemic, certainly in relation to the link between ZIKV and microcephaly ^21, 22, 31^.

Molecular xenomonitoring (MX) and xenosurveillance (XS) — pathogen detection in adult mosquitoes as a proxy for human infection — have been proposed as useful tools for VBD surveillance ^32– 35^. Often, routine *Ae. aegypti* surveillance at the community level involves the use of entomological indices such as the house, container, and Breteau indices (HI/CI/BI) that use abundance estimates of immature mosquito stages as a possible indication of disease risk. However, systematic, coordinated MX may prove a better and more appropriate tool for arboviral surveillance because by capturing the adult mosquito it could be possible to determine arboviral infection rates.

In 2015, a three-year MX project on LF commenced in order to aid LF elimination activities in the RMR ^36^. The MX project was designed to provide detailed entomological data in an LF endemic area subjected to MDA and explore human and mosquito infection transmission dynamics over a small geographic area, to be scaled up in future, in order to inform a larger MX surveillance system. In the first year, the MX project collected nearly 11,000 adult female *Cx. quinquefasciatus* and *Ae. aegypti* mosquitoes from July to August 2015.

The MX project had a primary objective of screening *Cx. quinquefasciatus* for *Wuchereria* ( *W.) bancrofti* to determine if LF transmission was ongoing, as well as a secondary objective of screening *Ae. aegypti* for DENV (as, by the time the project commenced in early 2015, ZIKV and CHIKV were not yet known to circulate in Brazil). However, in order to detect the L3 infective stage of *W. bancrofti,* as well as to detect DENV, all collected mosquitoes were stored at −80°C for RNA preservation; this incidentally facilitated additional screening for ZIKV and CHIKV. Consequently, the *Cx. quinquefasciatus* and *Ae. aegypti* samples from July-August 2015 represented the earliest existing mosquito collection from the 2015-16 ZIKV epidemic in the RMR that were stored for RNA preservation.

Six months after MX data collection, Ministry of Health (MoH) data indicated there were microcephaly cases surrounding the study area from where these mosquitoes were collected. Further, while *Ae. aegypti* was considered the principal vector of ZIKV, limited speculation began on the possible role of *Cx. quinquefasciatus* in the transmission of ZIKV in Brazil ^37^. Therefore, in addition to screening *Ae. aegypti* for ZIKV, DENV, and CHIKV, a sub-set of the *Cx. quinquefasciatus* specimens were also screened for ZIKV. This paper presents the results of screening *Ae. aegypti* for ZIKV, DENV and CHIKV and *Cx. quinquefasciatus* for ZIKV, as well as the detailed results on *Ae. aegypti* abundance and physiological status, as these may relate to arbovirus infection and transmission in the RMR in 2015.

## Methods

### Study site characteristics

**Figure 1.**
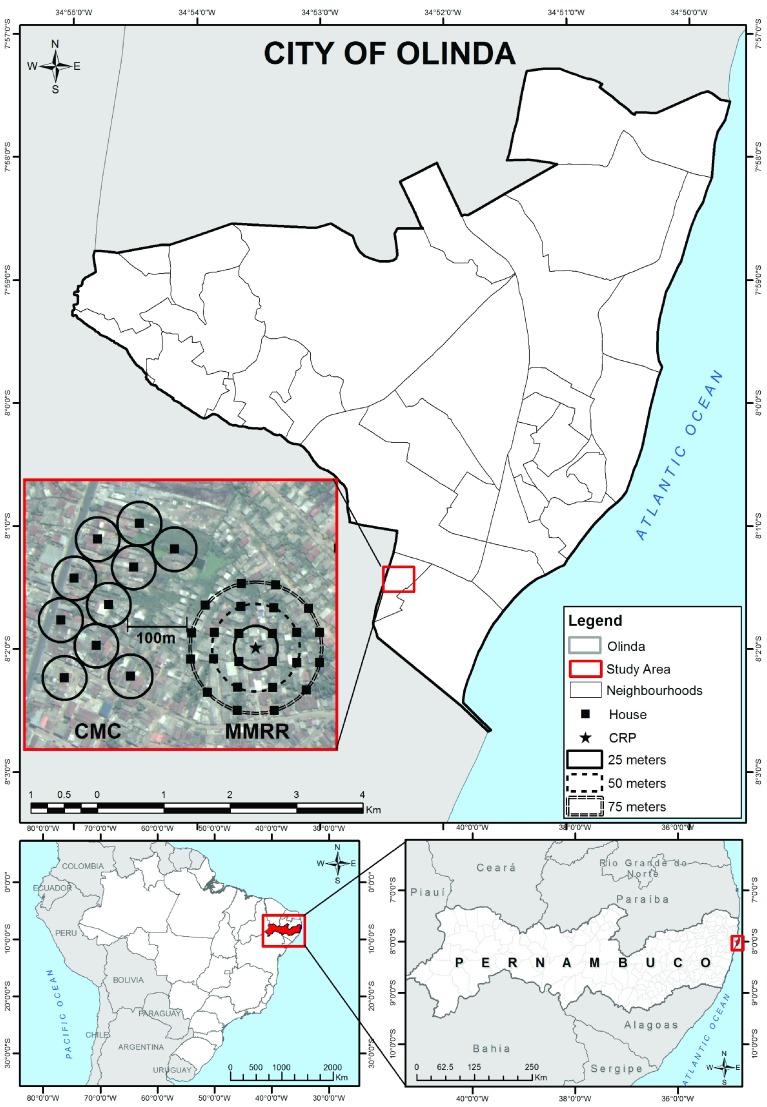
Map of the City of Olinda, RMR, Pernambuco State, Brazil and two study sites (collection method comparison (CMC) and mosquito mark release recapture (MMRR)) from a central release point (CRP) containing 35 sentinel mosquito collection points within Sítio Novo, Olinda (July 22-August 21, 2015). Maps display City of Olinda, situated within the state of Pernambuco and country of Brazil, as well as two study sites containing 35 sentinel mosquito collection points within Sítio Novo, Olinda consisting of (a) 10 houses in a CMC and (b) 25 houses in a MMRR from a CRP.

Olinda is the second most populous and population-dense city of the RMR, with 377,779 residents in its area of 41.68 km ^2^ ( Figure 1) ^27^. It has a tropical monsoon climate (Köppen climate classification = *As*), and temperatures range from 30°C (86°F) in January and February to 21°C (70°F) in July ^38^. The dry season peaks in November (average 36 mm rainfall), while the rainy season peaks in July (average 388 mm rainfall) ^39, 40^.

Mosquitoes screened in this study were collected from two sites within Sítio Novo, Olinda, RMR ( Figure 1). Between them and accounting for a 100 m buffer zone, the two study sites covered a combined area of approximately 0.4 km ^2^ containing approximately 5,514 residents, which was roughly equivalent to 1% of the territorial area and 1% of the population of Olinda, RMR ( Figure 1) ^27^. As previously described, the mosquitoes used in this study were captured from two studies to optimise an urban MX system for LF and arboviruses: a collection method comparison (CMC) to determine the ideal mosquito capture method and a mosquito mark release recapture (MMRR) study to determine mean and maximum mosquito dispersion ^36^. Mosquito collection occurred between July 22 and August 21, 2015, coinciding with the end of the rainy season and associated peak in mosquito abundance.

### House selection and mosquito collection

Detailed information on the study design and methods by which mosquitoes were collected have been described elsewhere ^36^. Briefly, houses were selected for participation from satellite images and geographic information systems (GIS) software of ArcGIS 10.2 (ESRI 2014. ArcGIS Desktop: Release 10. Redlands, CA: Environmental Systems Research Institute) and QGIS 2.10.1(QGIS Development Team (2015). QGIS Geographic Information System. Open Source Geospatial Foundation Project). Participant house selection accounted for geographic (aligning along transport arteries in CMC) and environmental (e.g., avoiding mangrove in MMRR) barriers, as well as local health authority advice on the most secure areas to work. A combination of global positioning system (GPS) devices (Garmin GPSmap 76cs, 3 m precision) and geographic information system (GIS) / satellite image maps were used to locate selected houses.

Mosquitoes were collected in 35 houses between the two study sites as follows: a) 10 houses from the CMC study comparing battery-powered hand-held aspirators with Centers for Disease Control and Prevention (CDC) light traps; and b) 25 houses from the MMRR study using fluorescent dust to detect mosquito dispersion (i.e., mean and maximum flight distance) using battery-powered hand-held aspirators only. The CMC study site was a commercial and residential zone with some paved streets, municipal sanitation, and drainage systems where houses were of higher quality construction, with brick walls, solid/permanent roofs, some partially screened windows, and fewer wall openings. The MMRR study site was an infrastructure-lacking residential area with poorly paved streets, sanitation, and drainage where houses were often flooded from an adjacent area of riverine mangrove. After consent was obtained, participating households allowed study teams to enter their houses for daily mosquito collection for a total of 15 minutes per house during the hours of 9:00-11:30 am, Monday to Friday, over four weeks.

### Post-collection mosquito transport, processing, and storage

Mosquito collection nets were placed in an open-top storage box and transported back to the Instituto Aggeu Magalhães (IAM/FIOCRUZ Pernambuco) Insectary within two hours of field collection. Upon arrival, nets were immediately placed in a −20°C freezer for at least 20 minutes to immobilize the mosquitoes. Mosquitoes were then removed from the freezer and placed on ice for morphological identification, sex determination, and assessment of female physiological status. Female mosquito specimens were placed in Eppendorf tubes (maximum of 50 per tube, separated by species), labelled per house per day, and stored at −80°C.

### Molecular processing and arboviral screening

All female *Ae. aegypti* specimens collected were included in molecular processing and arbovirus screening for ZIKV, DENV and CHIKV. For this species a pooling strategy of up to 10 mosquitoes per pool, grouped per house per week, was used. In addition, a sub-sample (15%) of female *Cx. quinquefasciatus* were screened for ZIKV, with the remainder preserved at −80°C for future molecular analysis. For *Cx. quinquefasciatus* specimens, a pooling strategy of up to 10 mosquitoes, grouped per house per day (MMRR) or per house per week (CMC) was used. Sample processing comprised RNA extraction, reverse transcription to generate cDNA, followed by PCR screening as detailed below. Prior to screening for arboviruses, each cDNA sample was tested using the appropriate mosquito species PCR to confirm RNA extraction and reverse transcription had been successful.

### RNA extraction

RNA was extracted from *Ae. aegypti* mosquito pools using a combined TRIzol ^®^-RNeasy ^®^ Mini kit extraction methodology *. Ae. aegypti* pools were homogenised on ice in 200 µL of nuclease-free water using a sterile plastic pestle (Sigma Aldrich) to homogenise by hand. Once fully homogenised, and while mixing by pipetting to ensure a homogeneous solution, 100 µL of each homogenate was aliquoted and stored at −80°C for the possibility of later virus isolation. To the remaining 100 µL of homogenate, 1 mL of TRIzol ^®^ (Ambion) reagent was added and mixed thoroughly by inversion before incubation for 5 minutes at room temperature. 200 µL of chloroform was added and each tube was shaken vigorously by hand for 15 seconds, before a further 3-minute incubation at room temperature. Samples were centrifuged (Heraeus Megafuge 8R, Thermo Fisher Scientific) at 12,000 x g for 15 minutes at 4°C for phase separation. The clear upper aqueous phase (~650 µL) containing the RNA was carefully taken off and placed on ice, and the lower phases were stored at −80°C for any future analysis. An equal volume (650 µL) of 70% ethanol was added to the aqueous phase on ice and mixed carefully by pipetting before immediately transferring up to 700 µL of the mixed sample to a labelled RNeasy ^®^ Mini spin column (QIAGEN), placed in a collection tube. The spin column was centrifuged at room temperature (21°C) for 15 seconds at 8,000 x g, the flow-through was discarded, and the remaining aqueous phase-ethanol mix was added to the spin column and the centrifugation process repeated. Once the samples had been added to the RNeasy ^®^ Mini spin columns, the manufacturer’s instructions were followed in subsequent steps according to the RNeasy ^®^ Mini procedure, with 700 µL of Buffer RW1, then 500 µL of Buffer RPE for each of the two subsequent wash steps. RNA was eluted from the spin columns in two separate elutions of 30 µL RNase-free water, with the first elution being used for reverse transcription to generate cDNA for downstream PCR analysis, and the second being stored at −80°C for any future analysis. RNA had been extracted previously from *Cx. quinquefasciatus* pools as part of the MX project for *W. bancrofti* screening using a TRIzol ^®^ extraction procedure ^36^.

### Reverse transcription

RNA samples were reverse transcribed using a QIAGEN QuantiTect ^®^ reverse transcription kit according to manufacturer’s instructions. Briefly, genomic DNA was removed by adding 2 μL gDNA Wipeout Buffer (7x) to 12 μL template RNA followed by incubation in a thermal cycler (GeneAmp™ PCR System 9700, Applied Biosystems™) at 42°C for 2 minutes, prior to immediately placing the reactions on ice. Next, 6 μL of reverse transcription master mix was then added to each sample, comprising 1 μL RT Primer Mix, 4 μL Quantiscript ^®^ RT Buffer (5x) and 1 μL Quantiscript ^®^ Reverse Transcriptase per sample. All reactions were prepared on ice. The final reactions were placed in the thermal cycler at 42°C for 30 minutes, followed by 95°C for 3 minutes before a hold at 4°C.

### Mosquito species real-time PCRs

In order to confirm successful generation of cDNA from mosquito samples, the appropriate mosquito species PCR was used to check each group of cDNA samples. For *Ae. aegypti,* a species-specific probe-based assay was used, targeting the internal transcribed spacer 1 (ITS1) region of this species ^41^. The primers and probe used were *Ae. aegypti* FOR (named ITS1_F338 in 41): 5’-CGCTCGGACGCTCGTAC-3’, Alternative *Ae. aegypti* REV: 5’-GGCGGCTTCGAGCTTC-3’ and *Ae. aegypti* Probe (AegyITS1P in 41): 5’-6-FAM-CAGAACACGCCAGACACGTTCGTACG-TAMRA-3’. The alternative reverse primer was an adjustment from the reverse primer (ITS1_R427) in 41 after alignment of *Ae. aegypti* ITS1 sequences available in GenBank at the time of preparatory work for the study, generated from source material from multiple countries and continents, highlighted genetic variation in the primer binding site for ITS1_R427. The alternative reverse primer was therefore designed, aiming to account for areas of genetic variation and improve detection of *Ae. aegypti* from different localities. As no ITS1 sequences for Brazilian *Ae. aegypti* were available at the time, a consensus sequence from those available for alignment was generated to try to best account for the genetic variation for use as a synthetic standard positive control. The 99 bp target consensus sequence used was 5’-CGCTCGGACGCTCGTACGTACCGCACCACAACCGCATCCGTACGAACGTGTCTGGCGTGTTCTGAACTGAACTGTGTCTCGCCGAAGCTCGAAGCCGCC-3’.

In addition, to cross-check for specificity of the *Ae. aegypti* PCR results from the cDNA samples in this study, an *Ae. albopictus* species-specific probe based PCR ^41^, also targeting the ITS1 region, was used on a small sub-set of the samples. The primers and probe used were *Ae. albopictus* FOR (named ITS1_F440 in [ 41]): 5’-GTCAGCAGGGCCGAACC-3’, *Ae. albopictus* REV (ITS1_R510 in [ 41]): 5’-GACGACCCGCCACTTAGCT-3’, *Ae. albopictus* Probe (AlboITS1P in [ 41]): 5’-6-FAM- CAGGGCACATACGTCCGCTTTGGTT-TAMRA-3’ and the 71 bp target sequence (5’-GTCAGCAGGGCCGAACCCGCGCAGGGCACATACGTCCGCTTTGGTTTGACATAGCTAAGTGGCGGGTCGTC-3’) was used to generate a synthetic standard to use as a positive control.

For both *Aedes* species-specific probe-based assays, PCR reactions were prepared using 10 μL of 2x Promega GoTaq ^®^ Probe qPCR Master Mix, a final concentration of 0.3 µM of each primer, 0.1 µM probe, 5µl of nuclease free water and 2 μL template cDNA to a final reaction volume of 20 μL. 2 μL CXR passive reference dye added to each 1 ml GoTaq Probe qPCR Master Mix tube on first use. Prepared reactions were run on an Applied Biosystems 7500 Fast Real-Time PCR System for 2 minutes at 95°C, followed by 40 cycles of 95°C for 15 seconds and 60°C for 1 minute. The increase in FAM fluorescence was monitored in real time by acquisition during the combined annealing/extension step of each cycle using the FAM filter. ROX was used as the passive reference dye. Synthetic standard positive controls (a minimum of 3, 10-fold dilutions per run), in addition to no template controls (NTCs) were included on each PCR run. Results were analysed using the 7500 Fast Software v2.0.6 and the inter-assay quantitation cycle (Cq) values produced by the synthetic standard positive controls were comparable across runs.

For cDNA generated from pools of *Cx. quinquefasciatus,* a host SYBR green quantitative PCR assay was designed targeting the S7 ribosomal protein (S7) mRNA gene (GenBank Accession # AF272670.1) ^36^. Primers used were *Cx. quinquefasciatus* FOR: 5’ -AAGGTCGACACCTTCACGTC-3’ and *Cx. quinquefasciatus* REV: 5’-GCGCCGCGAATAGTTTACAG-3’ and the 95 bp target sequence (5’-AAGGTCGACACCTTCACGTCGGTGTACAAGAAGCTGACCGGACGCGACGTCACGTTCGAGTTCCCGGAACCCTACCTGTAAACTATTCGCGGCGC-3’) was used to generate a synthetic standard to use as a positive control.

PCR reactions were prepared to a total volume of 25 μL, containing 12.5 μL of 2x QIAGEN QuantiTect ^®^ Sybr Green Master Mix, 0.3 µM final concentrations of each primer, 8.5 μL of nuclease-free water and 2 μL of template cDNA. Prepared reactions were run on an Applied Biosystems 7500 Fast Real-Time PCR system for 15 minutes at 95°C followed by 40 cycles of 94°C for 15 seconds, 55°C for 30 seconds, 72°C for 30 seconds, and a final melt curve analysis as produced by the 7500 software (95°C for 15 seconds, 60°C for 1 minute, continuous detection during ramping to 95°C for 30 seconds, followed by a final 60°C for 15 seconds). Generation of amplified product in each reaction was monitored in real time through acquisition of SYBR green fluorescence readings during the annealing step of each cycle using the SYBR filter and the dissociation of generated products was monitored during the melt curve segment using continuous detection between the second and third steps, using the same SYBR filter.

### Arbovirus real-time PCRs

ZIKV screening was undertaken using a hydrolysis probe-based real-time PCR targeting the envelope structural gene ^42^ with the primers and probes ZIKV FOR (named ZIKV 1086 in 42): 5’- CCGCTGCCCAACACAAG-3’, ZIKV REV (ZIKV 1162c in 42): 5’-CCACTAACGTTCTTTTGCAGACAT-3’, and ZIKV Probe (ZIKV 1107-FAM in 42): 5’-6-FAM-AGCCTACCTTGACAAGCAGTCAGACACTCAA-BHQ1-3’. A synthetic standard for use as a positive control was generated with the sequence 5’- CCGCTGCCCAACACAAGGTGAAGCCTACCTTGACAAGCAATCAGACACTCAATATGTCTGCAAAAGAACGTTAGTGG-3’, comprising the 77 bp target sequence and matching the Brazilian ZIKV sequences available in GenBank at the time of study preparation. A local Brazilian ZIKV isolate (ZIKV strain *H. sapiens*/Brazil/PE243/2015; GenBank accession number: KX197192) was also used as a biological positive control ^43^. PCRs were carried out using the Promega GoTaq ^®^ Probe qPCR Master mix as detailed above, except primer and probe final concentrations were 0.9 µM and 0.25 µM, respectively.

In addition, an alternative ZIKV probe-based real-time PCR was used for confirmatory testing ^44^, with primers and probes Goffart ZIKV FOR: 5’-CTTGGAGTGCTTGTGATT-3’, Goffart ZIKV REV: 5’-CTCCTCCAGTGTTCATTT-3’, and Goffart ZIKV Probe: 5’-6-FAM-AGAAGAGAATGACCACAAAGATCA-TAMRA-3’. The 187 bp target sequence 5’-CTTGGAGTGCTTGTGATTCTGCTCATGGTGCAGGAAGGGCTGAAGAAGAGAATGACCACAAAGATCATCATAAGCACATCAATGGCAGTGCTGGTAGCTATGATCCTGGGAGGATTTTCAATGAGTGACCTGGCCAAGCTTGCAATTTTGATGGGTGCCACCTTTGCGGAAATGAACACTGGAGGAG-3’ was used as a synthetic standard positive control for this assay. The Promega GoTaq ^®^ Probe qPCR Master mix was used as fully detailed above, except primer final concentrations were 0.9 µM and final probe concentration was 0.25 µM.

DENV screening was undertaken using a SYBR green real-time PCR with generic pan-DENV primers ^45^; Pan-DENV FOR: 5’-TTGAGTAAACYRTGCTGCCTGTAGCTC-3’ and Pan-DENV REV: 5’-GTRTCCCAKCCDGCNGTRTC-3’. A serial dilution of DENV-2 cDNA generated from Aag2 cells (infected with DENV-2 at MOI 0.5, 5 days post-infection) was used as a biological positive control for this assay and PCRs were carried out using the QIAGEN QuantiTect® Sybr Green Master Mix as detailed above, except primer final concentrations were 0.5 µM, and the annealing temperature used in thermal cycling was 60°C.

CHIKV screening was undertaken using a SYBR green real-time PCR targeting the E1 structural gene ^46^. Primers used were CHIKV FOR (named CHIK/E1/10367/+ in 46): 5’-CTCATACCGCATCCGCATCAG-3’ and CHIKV REV (CHIK/E1/10495/- in 46): 5’-ACATTGGCCCCACAATGAATTTG-3’. The 129 bp target sequence of the E1 gene (5’-CTCATACCGCATCCGCATCAGCTAAGCTCCGCGTCCTTTACCAAGGAAATAATATCACTGTGGCTGCTTATGCAAACGGCGACCATGCCGTCACAGTTAAGGACGCTAAATTCATAGTGGGGCCAATGT-3’) was used to generate a synthetic standard to use as a positive control. PCRs were carried out using the QIAGEN QuantiTect® Sybr Green Master Mix as detailed above (primer concentrations of 0.3 µM and 55°C annealing temperature).

### Data analysis

Mosquito collection data were double entered by two independent data entry staff, cleaned, and analysed with Stata 14 (StataCorp. 2015. *Stata Statistical Software: Release 14*. College Station, TX: StataCorp LP). Maps of abundance were generated using ArcGIS 10.2 (ESRI 2014. ArcGIS Desktop: Release 10. Redlands, CA: Environmental Systems Research Institute). Data from mosquito collection and sorting were compiled in Excel to construct strategies for mosquito pooling. Molecular results from each individual PCR were analysed using the 7500 Fast Software v2.0.6 prior to exporting and combining all the data for each PCR test, for each sample, within an Excel database.

### Ethical approval and consent

Study aims and methods were presented to head of households and verbal and written informed consent was sought; households were enrolled upon receipt of written informed consent. All names, addresses, and GPS coordinates of participating houses and residents were concealed from study staff apart from the principal investigator and study coordinator, both of whom held the linking keys. Field teams worked during the mornings of weekdays due to security concerns as well as to increase acceptability of daily aspiration or CDC light trap placement/net collection. Ethical approval was obtained from the Research Ethics Committees of the Instituto Aggeu Magalhães (IAM/FIOCRUZ) and the London School of Hygiene & Tropical Medicine (LSHTM) [CAAE: 44535515.0.0000.5190; LSHTM: 10276; 10185].

## Results

### Vector abundance and physiological status

**Figure 2.**
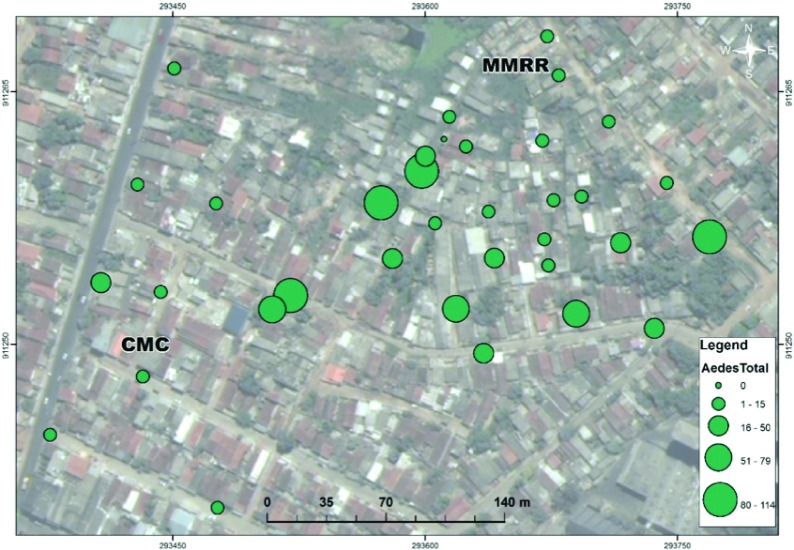
Spatial Distribution of Total *Ae. aegypti* (green) Female Mosquitoes in Sítio Novo, Olinda (July 22-August 21, 2015). Map displays mosquitoes captured July 22-August 21, 2015 from two study sites containing 35 sentinel mosquito collection points within Sítio Novo, Olinda consisting of (a) 10 houses in a collection method comparison (CMC) and (b) 25 houses in a mosquito mark release recapture (MMRR) from a central release point (CRP).

**Figure 3.**
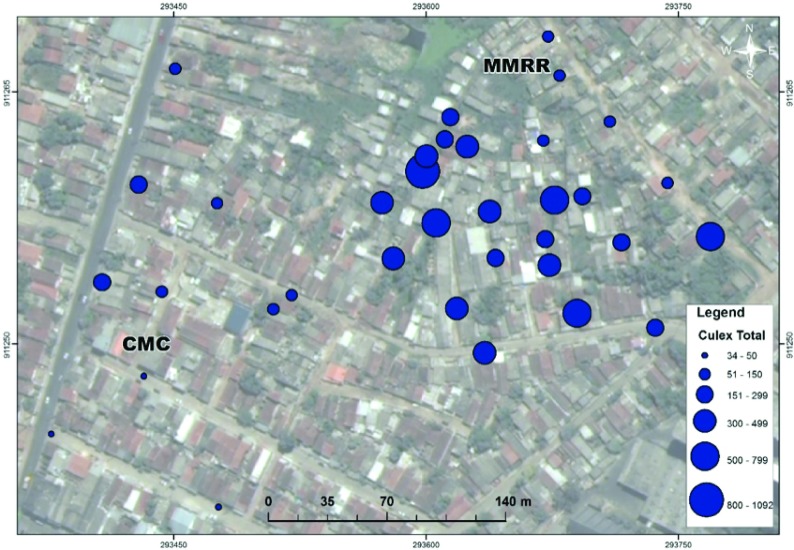
Spatial Distribution of Total *Cx. quinquefasciatus* (blue) Female Mosquitoes in Sítio Novo, Olinda (July 22-August 21, 2015). Map displays mosquitoes captured July 22-August 21, 2015 from two study sites containing 35 sentinel mosquito collection points within Sítio Novo, Olinda consisting of (a) 10 houses in a collection method comparison (CMC) and (b) 25 houses in a mosquito mark release recapture (MMRR) from a central release point (CRP).

A total of 11,078 adult female mosquitoes *(Ae. aegypti* and *Cx. quinquefasciatus*) were collected ( Table 1). As may be expected from studies that largely collected mosquitoes via battery-powered aspirators during the hours of 9:00-11:30 am, nearly 90% of the overall yield of mosquitoes were *Cx. quinquefasciatus*. Furthermore, as denoted in Table 1, nearly 65% of female mosquitoes collected were blood-fed, semi-gravid, or gravid. The spatial distribution of mosquitoes is presented in Figure 2 and Figure 3. *Ae. aegypti* displays a much more uniform spatial distribution across both study areas (CMC and MMRR, Figure 2). However, comparatively in space, *Cx. quinquefasciatus* is more aggregated within the area of poorest (MMRR) than the area of better (CMC) sanitation ( Figure 3).

**Table 1.** Adult Female *Cx. quinquefasciatus* and *Ae. aegypti* Collections, MX in Sítio Novo, Olinda (July 22-August 21, 2015).

Physiological Status	*Ae. aegypti*	*Cx. quinquefasciatus*	Totals
Unfed	116	3905	4,021
Blood-fed	348	4881	5,229
Semi Gravid	268	516	784
Gravid	207	837	1,044
Subtotal	939	10,139	11,078

### Molecular screening of *Ae. aegypti* and *Cx. quinquefasciatus* mosquitoes

As mentioned previously, 100% (939) of the *Ae. aegypti* and 15% (1,556) of the *Cx. quinquefasciatus* adult female specimens collected were included in molecular screening ( Table 2). All cDNA samples generated from mosquito pools, when tested with the respective ( *Ae. aegypti* or *Cx. quinquefasciatus*) species-specific PCRs, produced positive results. A small subset of the *Ae. aegypti* pools were also cross-checked with the *Ae. albopictus* PCR, giving all negative results. These species-specific results therefore demonstrated that each pool had been successfully processed to enable amplification by PCR and provided added confidence to the morphological identification and pooling of the specimens.

**Table 2.** The Number of Individual Specimens, Pools of Mosquitoes and Compositional Breakdown of Samples Included within Processing and Molecular Screening.

Physiological Status	*Ae. aegypti*	*Cx. quinquefasciatus*	Totals
Unfed	116	676	792
Blood-fed	348	724	1,072
Semi-Gravid	268	148	416
Gravid	207	8	215
Total individuals	939	1,556	2,495
Number of pools	156	182	338
Average pool size	6	9	7

All samples were initially tested in duplicate using the ZIKV PCR. Unfortunately, some widespread low-level amplification occurred with this assay, which appeared to be non-specific or produced equivocal results across the first and second tests. Any samples which appeared positive, or for which the initial results were inconclusive, were taken forward for a third test with the ZIKV PCR. Due to difficulties in generating unequivocal results using the original ZIKV PCR, confirmatory testing was carried out on a subset of those samples that remained inconclusive (including those demonstrating the strongest amplification previously) using the alternative Goffart ZIKV PCR. This assay produced negative results for all samples tested, with no amplification. Using a stringent process for analysis of the results, as no sample produced consistent, reliable and repeatable positive amplification on the original ZIKV PCR and the confirmatory testing demonstrated no amplification from any samples, it was concluded that all samples were negative for detectable ZIKV. All cDNA samples generated from the *Ae. aegypti* pools were also tested using the DENV and CHIKV PCRs but produced negative results. Cq values obtained from all PCR experiments are available as *Underlying data* ^47^.

## Discussion

This study, which screened *Ae. aegypti* mosquitoes for ZIKV, DENV, and CHIKV and *Cx. quinquefasciatus* for ZIKV, found no evidence of arboviral infection. However, the mosquitoes screened in this study were originally collected for the entirely different purpose of developing an MX system for LF elimination and so are subject to several points of discussion. The mosquitoes were sampled according to population-based designs appropriate for the development of an MX system for LF elimination. In particular, these designs sought a) to maximise numbers caught in a small area, while preferentially selecting those more likely to be infected (blood-fed, semi-gravid, and gravid), and b) to measure the spatial dispersion of *Cx. quinquefasciatus*, which again required a small study area ^36^. Hence, these designs were not powered to estimate the extent of infection in mosquitoes. This is a different approach to studies that are designed to collect mosquitoes from areas or populations with purposeful sampling methods related to high human infection prevalence (e.g., hospitals and houses with ZIKV-infected patients using index case methods), which would have been more likely to detect mosquitoes infected with arboviruses ^48^.

In general, infected mosquitoes would be most likely found in the same vicinity of infected human hosts (on whom mosquitoes are feeding). Additionally, the number of mosquitoes needed to detect infection in humans is inversely proportional, so in areas with a lower prevalence of human infection, more mosquitoes would be needed to detect infection and vice versa. However, in this case, the arboviral infection rates of the corresponding human population were not known at the time of collection (indeed, ZIKV and CHIKV were not known to circulate locally when the mosquitoes were collected in July-August 2015). Further, amongst reported arboviral disease from the time of mosquito collection, at the advent of circulating ZIKV and CHIKV in the RMR, there existed a high degree of misclassification based on physician diagnoses and a lack of laboratory-confirmed results to differentiate between the circulating arboviruses.

Mosquitoes screened in this study were collected from within a very limited geographic range, approximately 1% (0.4 km ^2^) of the territorial area and 1% (5,514 persons) of the population of Olinda, RMR ( Figure 1). In order to have a greater chance of capturing mosquitoes in an area of high or medium arboviral infection prevalence, it would have been ideal to capture mosquitoes over a wider geographic area, but the collection area was largely restricted due to logistical issues in running two concurrent field experiments. Unless the study site was an area of high human infection prevalence, it would have been very unlikely to detect infection in mosquitoes given the sample size of mosquitoes collected, and the geographic area over which they were collected. 

Mosquitoes screened in this study were collected at the time of peak mosquito abundance, within one month following the end of rainy season in the RMR ^39, 40^. However, peak arboviral incidence in the RMR typically occurs between November and March each year per regular Brazilian Ministry of Health reports; this has been largely established for DENV, although recent ZIKV and CHIKV epidemics seem to have affected these trends in recent years ^26, 49, 50^. Thus, while the study teams were able to collect a large quantity of mosquitoes over one month between July and August 2015, the likelihood of collecting and detecting arbovirus-infected mosquitoes would have always been expected to be low.

In accordance with the objective to design an MX surveillance system for the endgame of LF elimination in order to prevent or stem recrudescence, the original studies targeted *Cx. quinquefasciatus*. At the time, *Ae. aegypti* mosquito capture was a secondary objective. It should be mentioned that the absolute numbers of *Cx. quinquefasciatus*, including of potentially ‘exposed’ (blood-fed, semi-gravid, gravid) status, were notable in that a greater than expected number were captured; this may not be surprising given that battery-powered aspirators used in the CMC and MMRR experiments preferentially collect mosquitoes of all but unfed physiological status. The fact that fewer *Ae. aegypti* were collected than *Cx. quinquefasciatus* ( Table 1) is unsurprising given the collection method and deployment schedule, which were designed to heavily favour *Cx. quinquefasciatus* collection.

As aspirators preferentially collect post-bloodmeal resting females, and as aspiration occurred from 9:00-11:30 am each day in order to coincide with resting *Cx. quinquefasciatus*, it is not surprising that 90% of the overall mosquito yield was this species, ** as opposed *to Ae. aegypti.*

*Aedes aegypti* has long been established as the common vector for the flaviviruses DENV and ZIKV, as well as the alphavirus CHIKV (although in other regions, particularly Asia and SE Asia, *Ae. albopictus* is also considered a significant vector) ^51, 52^. Since 2016, there has been some speculation regarding the potential role of *Cx. quinquefasciatus* in ZIKV transmission ^37, 48, 53^. As such, this study screened *Ae. aegypti* (as would be expected, based on historical literature) for ZIKV, DENV, and CHIKV but also screened *Cx. quinquefasciatus* for ZIKV.

Since the mosquitoes screened in this study were collected, several studies have considered whether *Cx. quinquefasciatus* is capable of transmitting ZIKV ^44, 54– 67^; including research conducted in the RMR, although not specifically in Olinda ^37, 48^. Worldwide, since 2016, only two studies — one in Brazil and one in China — reported that *Cx. quinquefasciatus* may transmit ZIKV ^48, 53^. However, multiple other groups have not replicated these findings and cast doubt on this species’ vector competence for ZIKV. Since 2016, another group found that *Cx. quinquefasciatus* collected in China were not competent for ZIKV and recommended that *Ae. aegypti* be targeted for future vector control ^64^. Similarly, results from the RMR indicating that *Cx. quinquefasciatus* is competent for ZIKV transmission have not been replicated, and results from other studies have added weight to the theory that *Cx. quinquefasciatus* is not capable of transmitting ZIKV ^44, 54, 65, 66^.

As the present study was unable to detect arboviruses in *Cx. quinquefasciatus,* it can neither support nor confirm the potential role of *Cx. quinquefasciatus* in ZIKV transmission. It should be noted that even if infection had been detected in the *Ae. aegypti* or *Cx. quinquefasciatus* pools tested, it is possible that the mosquitoes could have simply ingested arbovirus-infected blood from a human host and were not involved in onwards transmission.

Low arboviral infection prevalence rates in field mosquitoes have resulted in difficulties in generating accurate estimates and often pooling is required for mosquito arboviral surveillance ^68– 74^. Additionally, publication bias may lead to negative studies being absent in the literature. Ultimately, a larger sample size of mosquitoes would have been needed to determine any correlation between mosquito infection rates and human cases in the RMR (but it should be noted that doing so was not the objective of the CMC and MMRR studies from where the mosquitoes tested in this study originated). For example, a recent study conducted in Puerto Rico reported CHIKV infection rates (IRs) of 1.75-2.48 infected mosquitoes/1000 mosquitoes, IRs for ZIKV of 1.2-2.0 infected mosquitoes/1000 mosquitoes and IRs for DENV of 0.15-0.67 infected mosquitoes/1000 mosquitoes resulting from more than 77,000 mosquitoes analysed ^75^.

The mosquito samples screened in this study are the earliest known adult mosquito samples from the RMR that were collected and stored for RNA virus preservation and analysis during the onset of the ZIKV outbreak. Nevertheless, the study team experienced significant intra- and inter-institutional delays with permissions, including significant logistical restrictions on exporting samples and with obtaining reagents, to some extent similar to those described by others in relation to this Zika outbreak ^76^. In addition to logistical considerations, there can often be difficulties in the rapid introduction of new molecular techniques in outbreak situations. During the initial stages of the discovery of a pathogen in a novel location, it can often take some time for genetic sequence data and information on any genetic variation of local isolates to become widely available. In such situations, previously established PCR methods are likely to have been designed using sequences from historical, geographically disparate isolates, increasing the potential difficulties in quickly identifying and adopting the most suitable PCR method to use. A lack of such sequence data for *in silico* molecular assay design and primer/probe binding checks, to indicate whether they are likely to be of sufficient sensitivity for the current local isolates, can be problematic ^77^.

Often, difficulties can arise even when a well-established PCR is first used in a new setting, particularly as part of a rapid outbreak investigation, where available reagents and equipment may vary. At the onset of an outbreak, there can also be limitations in availability of sufficient biological positive controls of local origin and from the appropriate sample matrix (in this case mosquitoes) for thorough sensitivity and specificity testing, optimisation, and quick and confident resolution of any equivocal results ^78^. For several such reasons, including the occurrence of non-specific amplification and equivocal results, which has been seen in some other studies, the analysis and interpretation of molecular results within this study has been highly stringent to avoid the possibility of false positive results ^77^. There remains a possibility that some variable amplification could occur due to the viral load present being on the limit of detection of an assay; however, as repeatable, reliable amplification was not obtained, our analysis concluded that no detectable ZIKV was found.

## Conclusions

None of the *Ae. aegypti* screened in this study revealed any evidence of arboviral infection with ZIKV, DENV, and CHIKV. Similarly, no *Cx. quinquefasciatus* pools were infected with ZIKV. Since mosquitoes were captured from an extremely restricted geographic area and during a very short time period of peak mosquito abundance (July to September), but low arbovirus circulation (November to March), this is perhaps not surprising. 

Yet, this research still identifies a role for continued MX of *Ae. aegypti* and *Cx. quinquefasciatus* for epidemic and emerging arboviruses in this densely populated urban setting. While this study did not detect arboviral infection in either species and the role of *Cx. quinquefasciatus* in ZIKV transmission has been largely refuted, it demonstrates that comprehensive MX may allow for retrospective, as well as prospective, analysis. MX also facilitates the testing of new hypotheses (including of divergent vector transmission pathways) and increases the chances of detecting emerging infections. In addition, opportunities for robustly testing collections of mosquitoes for multiple pathogens can provide greater possibilities for the generation of valuable pathogen transmission data, maximising the scientific output from the original sampling effort.

However, MX surveillance must be adjusted to allow for sufficient mosquito capture (particularly *Ae. aegypti* for arboviral surveillance), geographic coverage (encompassing greater environmental and population heterogeneity), and inclusion of different human populations potentially exposed to and at risk for pathogens under surveillance. It is important to note that from a public health risk assessment perspective, true MX would ideally be community-based in order to provide spatiotemporal estimates of infection and disease prevalence and incidence, as opposed to potentially biased estimates based on methods that preferentially screen those likely to be infected with pathogens of interest. Importantly, factors related to transmission dynamics should be prioritised when designing MX systems, including those related to both the mosquito (e.g. dispersion, species distribution, environmental factors such as availability of water for oviposition) and human (e.g. infection incidence, prevalence, host immunity to arboviruses) populations.

MX may seem labour and cost-intensive, and there is less data on studies using mosquitoes rather than humans to monitor VBDs in human populations. This is especially the case when considering the wealth of human health data that is readily available in most settings. However, while the current evidence base on MX may suggest that it is not yet refined enough to be used as a complete replacement or total proxy for monitoring VBDs in human populations, mosquito-based MX has the potential to greatly optimise current VBD surveillance and enhance early warning systems for of currently known and emerging VBDs in many parts of the world. In urban centres such as the RMR — where Zika (and its associated clinical sequale such as microcephaly and Guillain–Barré), dengue, chikungunya, and LF have caused enormous strain on public health systems in recent years — such enhanced surveillance systems could be very helpful for not only planning the allocation of public health resources but also better protecting the general public potentially affected by these devastating diseases. 

## Data availability

Open Science Framework: No evidence of Zika, dengue, and chikungunya virus infection in field-caught mosquitoes from the Recife Metropolitan Region, Brazil, 2015. https://doi.org/10.17605/OSF.IO/H2MJ9 ^47^.

This project contains all underlying real-time PCR data generated in this study.

Data are available under the terms of the Creative Commons Zero “No rights reserved” data waiver (CC0 1.0 Public domain dedication).
