# Supplementary material for: No evidence of Zika, dengue, or chikungunya virus infection in field-caught mosquitoes from the Recife Metropolitan Region, Brazil, 2015
Source: Wellcome Open Res. 2019 Jun 10;4:93. [Version 1] doi: 10.12688/wellcomeopenres.15295.1 (PMC6644828; doi:10.12688/wellcomeopenres.15295.1)
Supplement: Peer review history

# Peer review: Reviewer response for version 1

**DOI:** 10.21956/wellcomeopenres.16694.r35744

**Published:** 2019-07-19

**Authors:** Padet Siriyasatien

**Affiliations:** 1 Vector Biology and Vector Borne Disease Research Unit, Department of Parasitology, Faculty of Medicine, Chulalongkorn University, Bangkok, Thailand

Authors described the detection of Zika, dengue and chikungunya viruses in mosquitoes collected from endemic areas. Although, no virus detected in all samples but it would valuable as a base line data for future surveillance. The manuscript is well written in the flow of work and discussion. The only comment is to use italics for scientific name in the keywords.

I confirm that I have read this submission and believe that I have an appropriate level of expertise to confirm that it is of an acceptable scientific standard.

# Peer review: Reviewer response for version 1

**DOI:** 10.21956/wellcomeopenres.16694.r35837

**Published:** 2019-07-03

**Authors:** James Weger-Lucarelli

**Affiliations:** 1 Dept. of Biomedical Sciences and Pathobiology, College of Veterinary Medicine, Virginia Polytechnic Institute and State University, Blacksburg, VA, USA

The paper is well written and informative. The methods are clear and the authors used the correct techniques to try to answer the relevant research questions. The paper is probably more verbose than necessary which might obscure the important conclusion, that many mosquitoes were tested with no positives. I am well in favor of publishing these kinds of studies with "negative results" as I think it provides critical information that is needed to build a timeline of how an outbreak occurred.

I confirm that I have read this submission and believe that I have an appropriate level of expertise to confirm that it is of an acceptable scientific standard.
